# 

**Published:** 1917-02-10

**Authors:** 


### CONTENTS.

PAGE

**The Medical Profession and the War** ... ... 375

**On Guard in Wartime** ... ... ... ... 376

**Hospital and Institutional News** ... ... 377

The Great Central Hotel as a Clearing House for Wounded Officers—Lord Rhondda and Infant Welfare—A Many-sided Career—A Hospital Superintendent Receives the C.B.—A Long Career of Institutional Service—A Charge of False Pretences—Co-operators and the Royal Lancaster Infirmary—The York Workpeople's Fund—Is an Auditor's Report Confidential?—A Cottage Hospital and a Sacred Well—Children's Hospitals: A Partnership Scheme—Distraction for the Wounded—Burning Fatalities at West Bromwich—Fees for Hospital Ambulances—Parenthood and the Poor-Law Veto—The Plymouth Royal Eye Infirmary—Sectarian Bickering at the Metropolitan Asylums Board—This Week's Drug Market.

**Sir Alfred Keogh, G.C.B., M.D.** ... ... ... 381

The Director-General of the Army Medical Service.

**How German Hospital Ships are Fitted** ... 382

**Re-education in Walking** ... ... ... ... 383

By James B. Mennell, M.A., M.D., B.C.Cantab., etc.

**The New Electrical Department at Guy's** ... 385

A Veteran among Hospitals Keeping Up to Date.

**Leicester and County Hospital Saturday Society** ... ... ... ... ... ... 386

PAGE

**Patriotism in British Hospitals** ... ... ... 387

Two London Medical Schools.

**In Wartime** ... ... ... ... ... ... 388

An Eastern County Hospital's Preparations.

**The Matrons' and Sisters' Department** ... 389

Surprise Packets of Wartime.
Round the Hospitals.
The Devotion of English Nurses—A Curious Christmas—The New Matron at Guy's—The Scottish Board at Work—Miss Brunton's Appointment—Irish Questions and the College.

**The College and Ireland** ... ... ... ... 391

Further Questions and Answers.

**Matrons' and Sisters' Appointments** ... ... 392

**Passing Events and Topics...** ... ... ... 392

**Street Collections** ... ... ... ... ... 393

New Regulations of the Secretary of State.

**Independent Audit of Asylum Accounts** ... 394

**The Institutional Worker** ... ... (Suppl.) 1

Attendance Record for Casualty Cases: Answer to January Question.
The Ward Linen Supply: Answer to January Question.
Splints and the Metal Problem.
Question for February.
Enquiries and Answers.
The Sick and in Need.
Employment and Training.

